# Neuropsychological stability in classical galactosemia: A pilot study in 10 adult patients

**DOI:** 10.1002/jmd2.12410

**Published:** 2024-01-09

**Authors:** Merel E. Hermans, Gert J. Geurtsen, Carla E. M. Hollak, Annet M. Bosch

**Affiliations:** ^1^ Department of Pediatrics, Division of Metabolic Diseases Amsterdam UMC location University of Amsterdam, Emma Children's Hospital Amsterdam The Netherlands; ^2^ Amsterdam Gastroenterology Endocrinology Metabolism, Inborn Errors of Metabolism Amsterdam The Netherlands; ^3^ Department of Medical Psychology, Amsterdam Neuroscience, Neurodegeneration Amsterdam UMC location University of Amsterdam Amsterdam The Netherlands; ^4^ Department of Internal Medicine, Division of Endocrinology and Metabolism Amsterdam UMC location University of Amsterdam Amsterdam The Netherlands

**Keywords:** cognitive change, cognitive functioning, galactosemia, GALT‐deficiency, neuropsychology, progression

## Abstract

Classical galactosemia (CG) is an autosomal recessive disorder of galactose metabolism. Despite early initiation of a galactose‐restricted diet, patients develop long‐term complications including cognitive impairment. There is an ongoing debate whether the cognitive impairment in CG is stable throughout life or progresses with age. Earlier cross‐sectional and longitudinal studies regarding intelligence suggest stability, but longitudinal neuropsychological studies focusing on specific cognitive functions are limited. Therefore, the aim of this study is to assess cognitive change over time in adult CG‐patients. Ten adult patients with normal to borderline intelligence (mean age 33 years, range 22–49; IQ≥70 or independent work‐ or living situation) were assessed twice with a mean time interval of 3 years and 9 months (range 1023–1575 days). The neuropsychological assessments covered information processing speed, executive functioning, verbal fluency, and visuospatial functioning. Results showed that there was no significant decline or improvement in test scores on all neuropsychological measures except a decline on the Trail Making Test‐A (*p* = 0.048). However, this group‐level difference was subject to “regression to the mean” and was not endorsed by significant change in test scores measuring the same cognitive domain. Moreover, no specific pattern of reliable change (RCI > ‐1.96) was present on specific measures or within individual patients. This explorative study performed in 10 adult CG‐patients with normal to borderline intelligence revealed no cognitive change on several cognitive domains. This implies that the subset of adults with a normal to borderline IQ in their early and middle adulthood are cognitively stable.

**Synopsis**

This pilot study indicates that neuropsychological functioning is stable in adult patients with classical galactosemia and normal to borderline intelligence.

## 1 INTRODUCTION

Classical galactosemia (CG; OMIM 230400), caused by a deficiency of galactose‐1‐phosphate uridyltransferase (GALT; EC 2.7.7.12), leads to severe neonatal illness after the ingestion of galactose from breastmilk or formula in the first week of life. Timely start of the only available treatment, a lifelong galactose‐restricted diet, resolves the critical symptoms but does not prevent long‐term complications.^1^ Besides speech and language difficulties, motor disorders, and primary ovarian insufficiency (female patients), the majority of patients suffer from lower intellectual abilities and neuropsychological impairment.^2^, ^3^ Patients differ in severity and profile of the cognitive difficulties but impairments in attention and information processing speed, (working) memory, visuospatial functioning, cognitive flexibility and social cognition seem to be most prevalent.^4^, ^5^, ^6^, ^7^ These cognitive difficulties have a negative impact on the quality of life of CG‐patients.^8^

A recurrent question is whether the cognitive impairments in CG are stable throughout life or progress with age. This question has been mainly investigated by focusing on the total intelligence quotient (TIQ).^9^, ^10^, ^11^, ^12^ As previously described by Fridovich‐Keil and Berry,^13^ cross‐sectional and longitudinal studies together suggest that intellectual (dis)abilities remain stable over time in CG‐patients. In CG, two studies evaluated cognitive decline while focusing on specific cognitive functions.^14^, ^15^ Only one study used a longitudinal design assessing cognition with subtests of the Woodcock‐Johnson Tests of cognitive ability, measuring (short)term memory, auditory and visual processing, picture vocabulary, reading, spelling, and math, and the Beery Test of Visual Motor Integration in a sample of six children with CG. They did not find any differences on any of the tests after 2–5 years. Even though this finding suggest that the cognitive difficulties in CG are not progressive in children, the evidence is very limited. Therefore, longitudinal neuropsychological studies in CG‐patients are needed. The aim of the current retrospective longitudinal pilot study is to assess cognitive change over time in adult CG‐patients with a normal to borderline total intelligence.

## 2 METHODS

### 2.1 Patients and recruitment

All adult CG‐patients in our galactosemia expertise outpatient clinic with two known pathogenic *GALT*‐variations and/or an erythrocyte GALT activity <15% of healthy controls who received at least two standardized neuropsychological assessments using the same neuropsychological tests, were eligible for inclusion in this study. All eligible patients received the second evaluation in the context of our recent study of social cognition.^7^ Due to the inclusion criteria of this study, only CG‐patients with normal to borderline intelligence (Total IQ ≥70 and/or an independent work or living situation) were included in this study. Exclusion criterion was the presence of a second genetic diagnosis impacting clinical outcome. The Medical ethics committee of the AMC provided a waiver for this study. All data were collected after informed consent.

### 2.2 Procedure

The neuropsychological assessments were performed by a well‐trained neuropsychologist in either the hospital or during a home visit. Neuropsychological and medical data were retrospectively collected from medical files and the electronic clinical report forms in Castor Electronic Data Capture.^16^ In case of more than two neuropsychological assessments in a single patient, the two assessments with the most overlap in measures were selected.

### 2.3 Measures

The tests in the neuropsychological assessment are given in Table S1 and cover the following cognitive domains: Verbal and visual information processing speed, executive functioning (i.e., cognitive flexibility, inhibition), verbal fluency, and visuospatial functioning. See for the specific procedure of both neuropsychological assessments: Welsink‐Karssies et al.^5^ and Hermans et al.^7^ All raw scores were transformed to scaled scores (*T* scores: *μ* = 50, *σ* = 10) based on Dutch normative data. All normative data included a correction for age, except letterfluency.

### 2.4 Statistical analysis

First, patient characteristics were determined by means of descriptive analyses (i.e., age, gender, *GALT*‐variation, GALT erythrocyte activity, base of diagnosis, and IQ). Second, differences between the two neuropsychological assessments (T1 and T2) were evaluated by means of two‐sided paired‐sample *t*‐tests. A *p*‐value of <0.05 was deemed a statistically significant difference. Due to the small sample size in this pilot study, no correction was applied for multiple comparisons. Since the scaled scores for letterfluency were not automatically corrected for age, a correlation analysis was performed between age and both measurements of letterfluency. Third, the Reliable Change Index (RCI) was determined for each test in each patient to determine the changes were statistically reliable (Jacobson and Truax RCI >1.96) and to account for measurement error.^17^ The RCI was calculated according to (Testscore T2 – Testscore T1)/SE_diff_. SE_diff_ is the standard error of the difference between the testscores and is calculated using the standard deviation of the normative data (SD = 10) and the test–retest reliability. Finally, individual profiles were descriptively evaluated to assess the presence of clinically significant decline into (sub)clinical ranges (*T*‐score equal or below 36).

## 3 RESULTS

Ten adult patients with CG from our galactosemia expertise outpatient clinic were included. All patients received their first assessment in the context of patient care,^5^ and their second assessment in the context of our recent study of social cognition.^7^

The demographics of the patients are listed in Table 1. All patients had the classic phenotype of CG, defined as two pathogenic *GALT* mutations and absent or barely detectable erythrocyte GALT activity (<3.3%). All patients reported compliance to the galactose‐restricted diet at both assessment times. The mean time between the assessments was 1391 days (~3 years and 9 months; SD = 165.9 days, *Range* = 1023–1575 days). The time between measurement 1 and 2 was not related to the difference in standardized scores (results not shown).

**TABLE 1:** Demographics.

	*N*	CG‐patients (*N* = 10)
Gender, %		
Female	8	80%
Male	2	20%
Age at T1 in years, mean (range)	10	33.1 (22–49)
GALT erythrocyte activity (%), %		
<3.3	10	100%
≥3.3	0	0%
*GALT*‐variation, %		
p.Gln188Arg – p.Gln188Arg	6	60%
p.Gln188Arg – p.Lys285Asn	1	10%
p.Gln188Arg – p.Lys127Glu	1	10%
p.Gln188Arg – p.Glu172*	1	10%
p.Arg51Gln – p.Ser135Trp	1	10%
Diagnosis based on, %		
Clinical symptoms	7	70%
Family screening	3	30%
Total IQ, mean (range)	10	76.6 (69–83)

*Note*: *N*, sample size; CG, classical galactosemia; T1, time point 1; IQ, intelligence quotient.

### 3.1 Longitudinal group results

Group results are presented in Table 2. There was no significant decline or improvement in test scores on all measures except a small decline in speed on part A of the Trail Making Test, *t*(9) = −4.7, *p* = 0.048. This represents a delay of 3.5 seconds. Only 10% of the total of 80 difference scores were indicative of decline or improvement based on the RCI (see Table 2). Moreover, there was no specific pattern of change on specific tests and/or overarching cognitive domains (Table S2). There were no large differences in pattern of change between homozygous p.Gln188Arg‐patients and patients with other *GALT*‐variations, and between patients with a diagnosis based on clinical symptoms and patients with a diagnosis following family screening (i.e., with an immediate start of the diet; results not shown).

**TABLE 2:** Difference between the two neuropsychological assessments.

		*T* scores			RCI		
		Measurement 1	Measurement 2	*p*	Mean RCI (SD)	% reliable decline	% reliable improvement
Domain	Cognitive test	Mean (SD)	Mean (SD)				
Information processing speed							
WAIS‐IV Coding		40.0 (4.6)	41.4 (6.3)	0.404	0.21 (0.9)	0	0
Trail Making Test‐A		56.6 (6.8)	51.9 (6.5)	0.048*	−0.73 (1.0)	20	0
Stroop CWT – Word		46.7 (8.8)	46.8 (9.6)	0.953	0.02 (0.85)	0	0
Stroop CWT – Color		45.3 (9.3)	41.0 (12.9)	0.086	−0.84 (1.4)	20	10
EF—cognitive flexibility							
Trail Making Test‐B		49.1 (6.4)	49.5 (6.2)	0.865	0.09 (1.5)	0	10
EF—inhibition							
Stroop CWT—Color Word		47.3 (7.1)	45.2 (7.9)	0.301	−0.45 (1.3)	10	0
Verbal fluency							
Letterfluency		42.1 (11.1)	43.8 (8.6)	0.333	0.25 (0.8)	0	0
Visuospatial functioning							
GIT‐2 Spatial Test		36.3 (3.8)	38.5 (5.3)	0.256	0.42 (1.1)	0	10

*Note*: Results of the paired‐sample *t*‐tests between measurements 1 and 2 (*T* scores) together with the mean RCI and the proportion of patients with a reliable decline or improvement for each cognitive test.

Abbreviations: EF, executive functioning; GIT‐2, Groninger Intelligentie Test‐2; *p*, *p*‐value; RCI, Reliable Change Index; SD, standard deviation; Stroop CWT, stroop color word test; WAIS‐IV, Wechsler Adult Intelligence Scale‐IV.

* *p* < .05.

### 3.2 Individual profiles

The exploratory analysis of the individual patient profiles did not reveal any consistent change in scores and/or clinically significant decline (Table S2). No consistent change was present in both the patient subgroup performing in the average range on the first assessment and the patient subgroup performing in the (sub)clinical range (*T*‐score ≤ 36) on one of the tests at the first assessment. The two oldest patients (age above 45 years) did not demonstrate more change than the younger group (results not shown).

## 4 DISCUSSION

The current retrospective longitudinal study revealed no cognitive change in a small sample of adult patients with normal to borderline intelligence in a time period of ~3 years and 9 months (range 2–4 years). Specifically, verbal information processing speed, executive functioning (i.e., cognitive flexibility, inhibition), verbal fluency, and visuospatial functioning were not subjected to change. One of the measurements of visual information processing speed (i.e., Trail Making Test – part A) did show a group‐level difference between the first and second assessment. However, it is likely that these scores were subjected to the statistical phenomenon “regression to the mean”^18^ since both group assessment scores fell within the average range, and the only two individual patients showing a reliable decline had high scores on the first assessment. Furthermore, the other measurement of visual information processing speed (i.e., WAIS‐IV Coding) and the two verbal information processing speed measurements (i.e., Stroop CWT – Color naming and Word Reading) did not show a statistical difference between the two assessments. Therefore, we conclude that this group‐level difference is not statistical or clinically meaningful.

Our findings replicate the report on a small group of children with CG in the only previous longitudinal study addressing separate cognitive functions.^14^ The finding that no longitudinal change in cognitive functions is also present in adults suggests that CG is not a neurodegenerative disorder. Several pathophysiological mechanisms of the long‐term complications in CG have been proposed including very early damage from highly elevated metabolite (galactose‐1‐phosphate) levels in the fetal and newborn period,^19^ the toxic effects of the continuous endogenous production of galactose resulting in elevated levels of metabolites and the subsequent effect on galactosylation.^20^, ^21^ Since neuropsychological assessment is sensitive for changes in cognitive functioning due to neurodegeneration in an early stage,^22^ an ongoing pathophysiological process causing cognitive decline would likely have been detected by repeated neuropsychological assessment. Therefore, it is more likely that the cognitive impairment in CG originates in earlier developmental stages.^23^, ^24^ Importantly, the absence of cognitive decline in adults shows that the adults with normal to borderline intelligence in the current sample in their early and middle adulthood do not cognitively age faster than the general population. Since this study does not include CG‐patients with severe intellectual disabilities, no statements can be made about the cognitive aging of severely affected adult patients.

Despite the above‐described implications for CG, adult patients might still experience progressive cognitive complaints. As for all adults with concerns regarding cognition, referral to a neuropsychologist might help to establish whether these concerns belong to healthy or pathological cognitive aging. However, based on the findings of this study and previous reports on the stability of CG, repetitive neuropsychological assessments during early and middle adulthood in CG are not necessary.

### 4.1 Limitations

The main limitation of this study is the relatively small sample size of only 10 adult patients. Only a small subset of all CG‐patients received two neuropsychological assessments using the same neuropsychological tests, since repeated neuropsychological assessment is not part of standard patient care in adults.^25^ However, the current sample seems to be a well representation of the entire cohort of higher‐functioning CG‐patients in early‐ and middle adulthood in age, genetic variations and intelligence quotient. Because the small sample size consequently led to problems with statistical power, the individual profiles of the patients were evaluated using the RCI leading to the same results as for the group analyses. Also, no control group was included in this study. We compensated for this by using high‐quality normative data of the general population. All normative data except for one test (letterfluency) included a correction for age, making them suitable for distinguishing healthy cognitive decline due to aging and pathological cognitive decline.^26^ A last limitation of the study is the relatively short time period between the two assessments making it susceptible to small practice effects.^27^ Future studies might eliminate all practice effects by using a time interval of at least 5–7 years.

### 4.2 Strengths

This study is the first longitudinal study to investigate cognitive change in CG in a homogenous sample of adult patients. Moreover, the usage of neuropsychological tests facilitated the investigation of change in multiple cognitive domains in contrast to intelligence batteries and the subsequent report of only the TIQ.^26^ Intelligence test batteries are known for their resilience to age and brain injury,^26^, ^28^ and TIQ may obscure underlying vulnerabilities in specific cognitive functions. Therefore, neuropsychological tests are better suited to assess cognitive functioning and decline.^26^ Since for both assessments the same outcome measures were used, less bias was present since there were no differences in test procedures and the same normative data could be used to transform the raw scores to scaled scores.

## 5 CONCLUSION

The current pilot study indicates that CG‐patients in early and middle adulthood with normal to borderline intelligence demonstrate no cognitive change over time in the domains of information processing speed, executive functioning (i.e., cognitive flexibility and inhibition), visuospatial reasoning, and verbal fluency. The findings of this small study need to be replicated in larger, longitudinal studies with a larger time interval between measurements. However, the found absence of cognitive decline in this study, both on a group and individual level, adds to the current evidence that CG is not a progressive disorder.

## AUTHOR CONTRIBUTIONS

Merel E. Hermans administered the neuropsychological assessment, contributed to the study design, the data collection, the data analysis and interpretation, drafted the initial article, and critically revised the article. Gert. J. Geurtsen supervised the neuropsychological assessment and contributed to the study design, the data analysis and interpretation, drafted the initial article and critically revised the article. Carla E.M. Hollak contributed to the data collection and critically revised the article. Annet M. Bosch contributed to the study design, the data collection, the data analysis and interpretation, drafted the initial article, critically revised the manuscript, and serves as corresponding author for the article.

## FUNDING INFORMATION

This study was not funded by grants.

## CONFLICT OF INTEREST STATEMENT

Merel E. Hermans, Gert J. Geurtsen, and Annet M. Bosch declare that they have no conflict of interest. Carla E.M. Hollak declares that she is involved in premarketing studies of Sanofi and Idorsia.

## ETHICS STATEMENT

The local medical ethics committee gave a waiver for the data collection (reference numbers METC W18_100 no. 18.128 and W21_409 no. 21.456).

## PATIENT CONSENT STATEMENT

All procedures followed were in accordance with the ethical standards of the responsible committee on human experimentation (institutional and national) and with the Helsinki Declaration of 1975. The corresponding author confirms that all included patients gave informed consent for the use of their data for research purposes.

## Supporting information


**TABLE S1.** Overview of the neuropsychological assessment.

**TABLE S2.** Individual results.

## Data Availability

The data that support the findings of this study are available from the corresponding author upon reasonable request.
