# Supplementary material for: Neuropsychological stability in classical galactosemia: A pilot study in 10 adult patients
Source: JIMD Rep. 2024 Jan 9;65(2):110–5. doi: 10.1002/jmd2.12410 (PMC10910214; doi:10.1002/jmd2.12410)
Supplement: Supplementary file 1 — TABLE S1. Overview of the neuropsychological assessment. [file JMD2-65-110-s001.pdf]

Supplementary Table 1. Overview of the neuropsychological assessment.

**Cognitive domain**	**Measures**	**Norm**
Visual information processing speed	WAIS-IV Coding	A
	Trail Making Test (A)	A/E
Verbal information processing speed	Stroop Color Word Test (1-2)	A/G/E
Executive functioning – inhibition	Stroop Color Word Test (3)	A/G/E
Executive functioning – cognitive flexibility	Trail Making Test (B)	A/E
Verbal fluency	Letter fluency	E
Visuospatial functioning	GIT-2 Spatial Test	A

Notes. A = Corrected for age. E = Corrected for level of education. G = Corrected for gender. WAIS-IV = Wechsler Adult Intelligence Scale-IV. GIT-2 = Groninger Intelligentie Test-2.
