# Supplementary material for: Neuropsychological stability in classical galactosemia: A pilot study in 10 adult patients
Source: JIMD Rep. 2024 Jan 9;65(2):110–5. doi: 10.1002/jmd2.12410 (PMC10910214; doi:10.1002/jmd2.12410)
Supplement: Supplementary file 2 — TABLE S2. Individual results. [file JMD2-65-110-s002.pdf]

## Supplementary Table 2 – Individual results

| ID | Diff | Age | | WAIS-IV Coding | | | TMT-A | | | TMT-B | | | Stroop CWT – Word | | | Stroop CWT – Color | | | Stroop CWT – Color Word | | | Letterfluency | | | GIT-2 Spatial Test | | |
|---|---|---|---|---|---|---|---|---|---|---|---|---|---|---|---|---|---|---|---|---|---|---|---|---|---|---|---|
| | | T1 | T2 | T1 | T2 | RCI | T1 | T2 | RCI | T1 | T2 | RCI | T1 | T2 | RCI | T1 | T2 | RCI | T1 | T2 | RCI | T1 | T2 | RCI | T1 | T2 | RCI |
| 1 | 1575 | 26 | 30 | 50 | 47 | -0,55 | 67 | 54 | -2,01 | 52 | 46 | -1,28 | 61 | 61 | 0 | 43 | 56 | 2,55 | 51 | 56 | 1,07 | 67 | 64 | -0,44 | 28 | 40 | 2,27 |
| 2 | 1540 | 34 | 39 | 37 | 37 | 0 | 56 | 50 | -0,93 | 57 | 57 | 0 | 39 | 41 | 0,32 | 29 | 17 | -2,35 | 36 | 27 | -1,92 | 31 | 38 | 1,03 | 40 | 35 | -0,94 |
| 3 | 1506 | 31 | 35 | 40 | 37 | -0,55 | 56 | 44 | -1,85 | 44 | 58 | 2,98 | 52 | 48 | -0,65 | 56 | 55 | -0,2 | 54 | 50 | -0,85 | 31 | 38 | 1,03 | 40 | 45 | 0,94 |
| 4 | 1491 | 39 | 43 | 40 | 40 | 0 | 52 | 50 | -0,31 | 46 | 41 | -1,07 | 52 | 58 | 0,97 | 50 | 48 | -0,39 | 47 | 47 | 0 | 37 | 43 | 0,88 | 35 | 35 | 0 |
| 5 | 1472 | 28 | 32 | 40 | 40 | 0 | 53 | 54 | 0,15 | 54 | 48 | -1,28 | 56 | 54 | -0,32 | 53 | 45 | -1,57 | 53 | 46 | -1,49 | 40 | 44 | 0,59 | 40 | 35 | -0,94 |
| 6 | 1383 | 22 | 26 | 43 | 50 | 1,06 | 67 | 54 | -2,01 | 52 | 51 | -0,21 | 49 | 53 | 0,65 | 41 | 38 | -0,59 | 56 | 44 | -2,56 | 55 | 47 | -1,18 | 40 | 50 | 1,89 |
| 7 | 1364 | 47 | 51 | 33 | 40 | 1,28 | 48 | 51 | 0,46 | 41 | 40 | -0,21 | 35 | 30 | -0,81 | 31 | 21 | -1,96 | 37 | 44 | 1,49 | 43 | 47 | 0,59 | 35 | 40 | 0,94 |
| 8 | 1299 | 31 | 35 | 43 | 50 | 1,28 | 62 | 64 | 0,31 | 47 | 55 | 1,71 | 44 | 36 | -1,3 | 54 | 45 | -1,77 | 50 | 52 | 0,43 | 42 | 47 | 0,74 | 35 | 35 | 0 |
| 9 | 1260 | 24 | 27 | 37 | 43 | 0,90 | 56 | 57 | 0,15 | 58 | 51 | -1,49 | 35 | 44 | 1,46 | 48 | 43 | -0,98 | 48 | 47 | -0,21 | 39 | 37 | -0,29 | 35 | 35 | 0 |
| 10 | 1023 | 49 | 52 | 37 | 30 | -1,28 | 49 | 41 | -1,23 | 40 | 48 | 1,71 | 44 | 43 | -0,16 | 48 | 42 | -1,18 | 41 | 39 | -0,43 | 36 | 33 | -0,44 | 35 | 35 | 0 |

ID = Patient identification nummer. Diff = Difference in days between two neuropsychological assessments (T1 and T2). T1 = First neuropsychological assessment. T2 = Second neuropsychological assessment. RCI = Reliable Change Index. WAIS-IV = Wechsler Adult Intelligence Scale-IV. TMT = Trail Making Test. Stroop CWT = Stroop Color Word Test. GIT-2 = Groninger Intelligentie Test-2.
